# Racial and ethnic disparities in opioid use for adolescents at US emergency departments

**DOI:** 10.1186/s12887-021-02715-y

**Published:** 2021-05-31

**Authors:** Michael T. Phan, Daniel M. Tomaszewski, Cody Arbuckle, Sun Yang, Candice Donaldson, Michelle Fortier, Brooke Jenkins, Erik Linstead, Zeev Kain

**Affiliations:** 1 Chapman University, School of Pharmacy, Irvine, CA 92618 USA; 2 Department of Pharmaceutical & Health Economics, University of Southern California, School of Pharmacy, University Park Campus, 635 Downey Way, Bldg. #331, Los Angeles, CA 90089 USA; 3 Schmid College of Science and Technology, Chapman University, Orange, CA 92866 USA; 4 Irvine School of Medicine, University of California, Irvine, CA 92617 USA; 5 Center on Stress & Health, University of California School of Medicine, Irvine, USA; 6 Department of Anesthesiology and Perioperative Care, University of California, Irvine, USA; 7 Department of Pediatric Psychology, Children’s Hospital of Orange County, Orange, CA USA; 8 Sue & Bill Gross School of Nursing, University of California, Irvine, CA USA; 9 Department of Psychological Science, University of California, Irvine, CA USA; 10 Department of Psychology, Chapman University, Orange, CA 92866 USA; 11 Department of Pediatrics, CHOC Children’s, Orange, CA USA; 12 Yale Child Study Center, Yale University, New Haven, CT USA

**Keywords:** Pediatrics, Adolescents, Emergency department, Ethnology, Analgesics, Pain, National, Young adult, Minority, Race

## Abstract

**Background:**

Racial/ethnic disparities in the use of opioids to treat pain disorders have been previously reported in the emergency department (ED). Further research is needed to better evaluate the impact race/ethnicity may have on the use of opioids in adolescents for the management of pain disorders in the ED.

**Methods:**

This was a cross-sectional study using data from the National Hospital Ambulatory Medical Care Survey from 2006 to 2016. Multivariate models were used to evaluate the role of race/ethnicity in the receipt of opioid agonists while in the ED. All ED visits with patients aged 11–21 years old were analyzed. Races/ethnicities were stratified as non-Hispanic Whites, non-Hispanic Blacks, and Hispanics. In addition to race, statistical analysis included the following covariates: pain score, pain diagnosis, age, region, sex, and payment method.

**Results:**

There was a weighted total of 189,256,419 ED visits. Those visits involved 109,826,315 (58%) non-Hispanic Whites, 46,314,977 (24%) non-Hispanic Blacks, and 33,115,127 (18%) Hispanics, with 21.6% (95% CI, 21.1%-22.1), 15.2% (95% CI, 14.6–15.9%), and 17.4% (95% CI, 16.5–18.2%) of those visits reporting use of opioids, respectively. Regardless of age, sex, and region, non-Hispanic Whites received opioids at a higher rate than non-Hispanic Blacks and Hispanics. Based on diagnosis, non-Hispanic Whites received opioids at a higher rate in multiple pain diagnoses. Additionally, non-Hispanic Blacks and Hispanics were less likely to receive an opioid when reporting moderate pain (aOR = 0.738, 95% CI 0.601–0.906, aOR = 0.739, 95% CI 0.578–0.945, respectively) and severe pain (aOR = 0.580, 95% CI 0.500–0.672, aOR = 0.807, 95% CI 0.685–0.951, respectively) compared to non-Hispanic Whites.

**Conclusions:**

Differences in the receipt of opioid agonists in EDs among the races/ethnicities exist, with more non-Hispanic Whites receiving opioids than their minority counterparts. Non-Hispanic Black women may be an especially marginalized population. Further investigation into sex-based and regional differences are needed.

**Supplementary Information:**

The online version contains supplementary material available at 10.1186/s12887-021-02715-y.

## Background

Racial and ethnic inequities occur in a variety of aspects of our healthcare system in both adults and pediatrics [1]. In the emergency department (ED), Non-Hispanic Blacks and Hispanics wait significantly longer to receive care [2–4], have lower hospital admission rates [5], receive less diagnostic testing to evaluate chest pain [6], and receive fewer opioids to treat pain than Non-Hospanic Whites [7–9]. Although other studies have not found significant differences in pain management based on race and ethnicity [10–13], these studies have assessed all pain medications and not specifically the receipt of an opioid agonist. Additionally, studies have demonstrated that racial minorities, particularly non-Hispanic Blacks, report more pain than non-Hispanic Whites, while simultaneously receiving fewer opioid agonists during those visits [14, 15]. Disparities in pain management may have an adverse effect on minoritiy groups by creating significant barriers to obtaining quality health care, diminishing quality of life, and increasing societal costs.

Compared to the adult population, there is limited research in pain management disparaties among pediatrics, particularly in assessing the receipt of opioids in EDs. The use of opioids to treat pediatrics with moderate to severe pain is especially beneficial in moderate to severe pain that does not respond to acetaminophen and nonsteroidal anti-inflammatory drugs [16, 17]. Yet, opioid use has come under increased scrutiny in this population, resulting in decreased use in pediatrics within the ED. [3, 18]

Further complicating the discussion surrounding the use of opioids in pediatric patients is the reporting that opioid-related deaths among pediatrics aged 0–19 years have increased in recent history [19]. Within pediatrics, adolescents have been shown to be prescribed more opioids than pre-adolescent patients [3]. The use of opioids is especially concerning in adolescent patients as a whole, as recent studies suggest the prevalence of opioid misuse is high within this age group; however, previous studies have not suggested that Non-Hispanic Blacks or Hispanics have higher rates of opioid misuse [20–22]. In addition, research has suggested that opioid exposure in adolescence significantly increases the risk of subsequent opioid misuse or abuse later in life, which is also likely to encourage further restrictions on the use of opioids in this patient population [23, 24]. Although the risks associated with such potential for misuse is concerning, there are growing concerns over whether these concerns are resulting in the expansion of opioid prescribing disparities for Non-Hispanic Blacks and Hispanics, compared to their Non-Hispanic White counterparts [25]. Prescribers must find a balance between the conflicting risk of potential undertreatment and the potential adverse consequences of opioid use and ensure prescribing decisions are disconnected from any potential racial/ethnic bias. Managing pain must be driven by patient needs, based on clinical evaluation, without influence from patient specific demographic factors such as race/ethnicity.

Previous work conducted by Tomaszewski and colleagues found that Non-Hispanic White pediatric patients had received opioid agonists in EDs at a higher rate than Non-White pediatric patients in EDs, in addition to other non-clinically-driven prescribing patterns [3]. The existence of racial/ethnic inequalities in receipt of opioid agonists in EDs suggests the need to conduct a deeper examination of racial/ethnic disparities and whether they can be explained by other factors including geographic region, payment method, pain diagnosis, age, sex, episode of care, metropolitan setting, or time period. To date, no research has evaluated the impact of potential confounding clinical (pain score and pain diagnosis) and non-clinical factors (region of care, sex, age, and payment method) on pediatric opioid prescribing. Understanding the presence and degree of the racial/ethnic differences in the context of these factors provides insight on national opioid prescribing patterns regarding inequalities of healthcare. This study aims to further evaluate how these factors are associated with racial/ethnic differences in the receipt of opioid agonists among adolescents in EDs and their trends over time.

## Methods

### Study design and setting

This study analyzed data collected from the Center for Disease Control (CDC) and Prevention’s 2006–2016 National Hospital Ambulatory Medical Care Survey (NHAMCS). NHAMCS is conducted nationally among all 50 states and the District of Colombia using a representative sample of visits to hospital-based outpatient clinics, emergency departments, and ambulatory surgery locations in non-institutional and short-stay hospitals. The survey employs a complex four-stage study design. A detailed description of the data collection methodology can be accessed at the CDC’s National Center for Health Statistics (NCHS) website [26, 27].

### Population

Emergency department visits involving patients aged 11–21 years were included in the study and the study population was stratified into age groups of 11–14, 15–17, and 18–21 years old. This stratification was based on the American Academy of Pediatrics’ definitions of early, middle, and late adolescence [28].

### Outcome

The primary outcome was receipt of an opioid agonist in the ED. Drugs are coded in terms of generic component and therapeutic classification using Lexicon Plus®, a comprehensive database of medications available in the U.S. market. The usage of opioids was determined using the CDC’s New Ambulatory Care Drug Database system, with drugs being classified as opioid or other [29].

### Independent variables

NHAMCS categorized ethnicity as Hispanic and non-Hispanic. Race was categorized by White, Black, American Indian/Alaska Native, Asian, Native Hawaiian/Other Pacific Islander, or Multiple Races. Accordingly, we constructed 3 cohorts: non-Hispanic white, non-Hispanic Black, and Hispanic (Hispanic White and Hispanic Black). All other races were not included in our analysis since the resulting cohort sizes were lower than the CDC’s recommended number of visits per cohort. To examine the disparities of care for minorities, we used non-Hispanic White patients as the control group.

When recording race/ethnicity, hospital staff were instructed by NHAMCS to not ask the patient to self-identify their race/ethnicity, unless it was hospital procedure to do so. Therefore, it is likely race/ethnicity assigned to each patient reflects a clinician’s perception of the patient’s race/ethnicity, and not necessarily what classification the patient identifies with the most. Patient age, sex, payment method (private, Medicaid, self-pay, other payment method) were recorded at each visit. Information on whether the episode was an initial visit or follow-up visit was also collected. Healthcare institution information was also collected to determine region (Midwest, Northeast, South, West), and whether it was in a metropolitan setting or not.

The NHAMCS uses a standard reason for visit classification to code complaints, symptoms, or other reasons for visit. The summary of codes and diagnoses can be found in the NHAMCS micro-data file documentation [30]. Reported pain diagnosis were maintained as individual variables except for muscoloskeletal pain, which included arthritis/joint pain, pelvic pain, back pain, and neck pain. Pain was coded on a 1–4 scale, where a score of 1 meant no pain, 2 indicated mild pain, 3 reflected moderate pain, and 4 signaled severe pain [30]. A score of 0 in NHAMCS meant no pain score was recorded.

### Statistical analysis

All analyses were conducted on weighted data, as recommended by the CDC’s NCHS website. The weighting is calculated using the most recent census data to provide a stratified representation of the national patient population. All participants’ records were stored in a relational database using the open-source database software MySQL (v. 5.7.11, Oracle, Redwood Shores, California). All analytics were performed using the open-source statistical computing software R (v 3.2.3, R Foundation, Vienna, Austria). The function svydesign from the R package survey was used to account for stratified, clustered, and weighted variables in the NHAMCS data. Wald tests of association were used to determine significance for bivariate analyses. Stepwise regression via backward elimination was used including all independent variables mentioned above. Separate independent logistic regression models were run holding pain score constant with race/ethnicity as the sole independent variable and receipt of opioid as the dependent variable. CDC detailed documentation of the NHAMCS instrument, methodology and data files that were used as the basis for these analyses are available elsewhere [31].

## Results

### Patient characteristics and overall trend

From 2006 through 2016 there was a weighted total of 189,256,419 ED visits involving patients aged 11–21 years at the time of visit with a reported race/ethnicity of non-Hispanic Black, non-Hispanic White, or Hispanic. Those visits involved 46,314,977 (24%) non-Hispanic Black, 33,115,127 (18%) Hispanic, and 109,826,315 (58%) non-Hispanic White patients, with 15.2% (95% CI, 14.6–15.9%), 17.4% (95% CI, 16.5–18.2%), and 21.6% (95% CI, 21.1–22.1%) of those visits associated with receipt of an opioid, respectively (Table 1). Receipt of an opioid across all races/ethnicities was greater during the 2006–2011 timeframe compared to 2012–2016 (Table 1). In both time periods, non-Hispanic Whites received an opioid more frequently than non-Hispanic Blacks. However, compared to Hispanics, higher rates of opioid receipt was reported in non-Hispanic Whites in the 2006–2011 timeframe, but not 2012–2016.

**Table 1:** US emergency department visits for pediatric patients (2006–2016)^a^

Variable	Race/ ethnicity	Total visits weighted	Total received opioid weighted	Percent received opioids (weighted CI 95)^b^	Significance
All Visits	non-Hispanic Black	46,314,977	7,060,196	15.2% (14.6–15.9%)	TRUE
	Hispanic	33,115,127	5,744,969	17.4% (16.5–18.2%)	TRUE
	non-Hispanic White	109,826,315	23,740,585	21.6% (21.1–22.1%)	–
Age					
11–14	non-Hispanic Black	10,357,118	871,910	8.4% (7.3–9.5%)	TRUE
	Hispanic	9,835,028	1,065,055	10.8% (9.5–12.1%)	TRUE
	non-Hispanic White	27,210,118	3,875,138	14.2% (13.4–15.1%)	–
15–17	non-Hispanic Black	11,530,230	1,483,259	12.9% (11.6–14.1%)	TRUE
	Hispanic	8,476,686	1,267,072	15.0% (13.4–16.5%)	TRUE
	non-Hispanic White	28,845,980	5,411,450	18.8% (17.8–19.7%)	–
18–21	non-Hispanic Black	24,427,629	4,705,027	19.3% (18.2–20.3%)	TRUE
	Hispanic	14,803,413	3,412,842	23.1% (21.7–24.5%)	TRUE
	non-Hispanic White	53,770,217	14,453,997	26.9% (26.1–27.7%)	–
Metropolitan Setting					
Metropolitan Area	non-Hispanic Black	38,071,587	5,810,872	15.3% (14.5–16.0%)	TRUE
	Hispanic	27,392,274	4,773,270	17.4% (16.5–18.3%)	TRUE
	non-Hispanic White	76,616,787	17,818,822	23.3% (22.6–23.9%)	–
Non-Metropolitan Area	non-Hispanic Black	4,425,515	694,287	15.7% (12.8–18.5%)	FALSE
	Hispanic	2,084,413	340,727	16.4% (12.4–20.3%)	FALSE
	non-Hispanic White	23,766,791	4,322,886	18.2% (17.1–19.3%)	–
Unknown/Missing Data	non-Hispanic Black	3,817,875	555,037	14.5% (12.2–16.9%)	FALSE
	Hispanic	3,638,440	630,972	17.3% (14.8–19.9%)	FALSE
	non-Hispanic White	9,442,737	1,598,877	16.9% (15.3–18.5%)	–
Episode Of Care					
Initial visit to this ED	non-Hispanic Black	36,540,026	5,541,337	15.2% (14.4–15.9%)	TRUE
	Hispanic	26,760,576	4,560,584	17.0% (16.1–18.0%)	TRUE
	non-Hispanic White	87,184,961	18,229,727	20.9% (20.3–21.5%)	
Follow-up visit to this ED	non-Hispanic Black	2,100,319	406,880	19.4% (16.0–22.7%)	TRUE
	Hispanic	1,535,101	304,995	19.9% (16.0–23.7%)	TRUE
	non-Hispanic White	4,612,751	1,227,153	26.6% (24.0–29.2%)	–
Unknown/Missing Data	non-Hispanic Black	7,674,632	1,111,979	14.5% (13.0–16.0%)	TRUE
	Hispanic	4,819,450	879,390	18.3% (16.3–20.2%)	TRUE
	non-Hispanic White	18,028,603	4,283,705	23.8% (22.5–25.0%)	–
Year of Visit					
2006–2011	non-Hispanic Black	26,272,954	4,547,058	17.3% (16.4–18.2%)	TRUE
	Hispanic	16,352,926	3,198,892	19.6% (18.4–20.7%)	TRUE
	non-Hispanic White	61,622,253	15,602,711	25.3% (24.6–26.0%)	–
2012–2016	non-Hispanic Black	20,042,023	2,513,138	12.5% (11.5–13.6%)	TRUE
	Hispanic	16,762,201	2,546,077	15.2% (13.9–16.5%)	FALSE
	non-Hispanic White	48,204,062	8,137,874	16.9% (16.1–17.7%)	–

^a^All analyses accounts for the complex sampling design of NHAMCS

^b^Tests are run at 95% Confidence intervals (*P* < 0.05)

### Demographic factors

Across all age stratifications, non-Hispanic Whites were prescribed more opioids than minority patients (Table 1). Hispanics also tended to be prescribed more opioids than non-Hispanic Blacks, with statistical significance seen in the early- and late-adolescent ages. The largest gap observed was between early-adolescent non-Hispanic Blacks and non-Hispanic Whites, where opioid receipt was reported in 8.4% (95% CI, 7.3–9.5%) and 14.2% (95% CI, 13.4–15.1%) of visits, respectively.

**Fig. 1:**
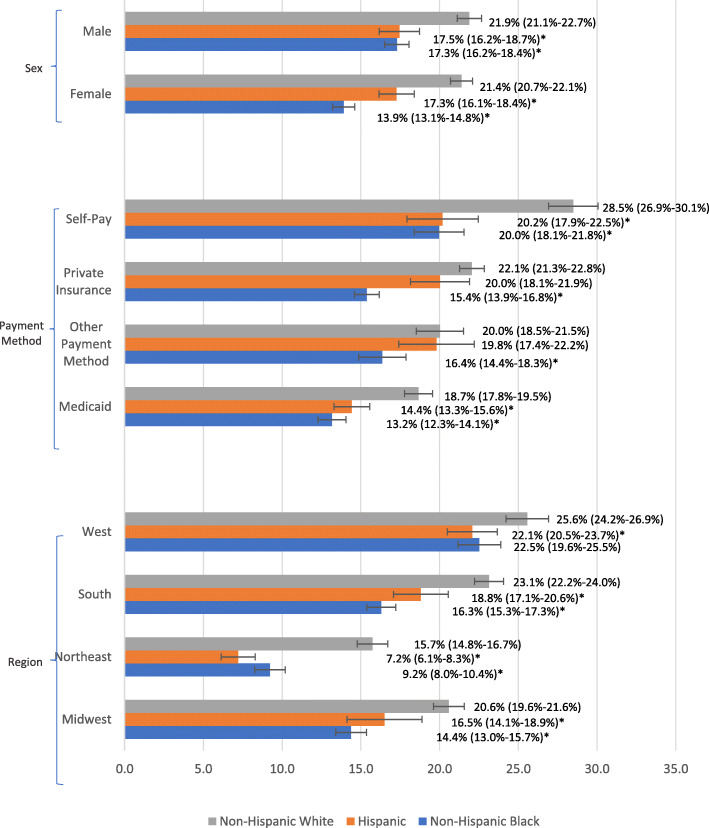
Opioid Prescription Rates by Demographic Factors ^a^. a: All analyses accounts for the complex sampling design of NHAMCS. Tests are run at 95% Confidence intervals (*P* < 0.05). * denotes statistical significance when compared to non-Hispanic White

Comparing racial/ethnic ED opioid receipt based on sex resulted in non-Hispanic White males and females having greater rates of opioid receipt reported than their Hispanic and non-Hispanic Black counterparts (Fig. 1). Visits with non-Hispanic White males and females reported opioid receipt in 21.9% (95% CI, 21.1–22.7%) and 21.4% (95% CI, 20.7–22.1%) of visits, respectively; whereas visits with non-Hispanic Black males and females reported receipt of an opioid in 17.3% (95% CI, 16.2–18.4%) and 13.91% (95% CI,13.1–14.8%) of visits, respectively. Visits with Hispanic males and females reported opioid receipt in 17.5% (95% CI, 16.2–18.7%) and 17.3% (95% CI, 16.1–18.0%) of visits, respectively.

Based on regions, non-Hispanic Whites were significantly prescribed more opioids than minorities in all regions, with the exception of the West. There, non-Hispanic Whites were prescribed more opioids than non-Hispanic Blacks, but not Hispanics (Fig. 1). The largest disparity was observed in the Northeast, where 15.7% (95% CI, 14.8–16.7%) of non-Hispanic Whites, 9.2% (95% CI, 8.0–10.4%) of non-Hispanic Blacks, and 7.2% (95% CI, 6.1–8.3%) of Hispanics had reported receipt of an opioid within the ED. The Midwest and South reported a similar pattern. Overall opioid use within the ED appears to be highest in the West and lowest in the Northeast (Fig. 1). An evaluation of opioid use based on whether the hospital was located within a metropolitan or non-metropolitan area are found in Table 1.

Race/ethnicity differences were also observed in payment method, where ED visits with non-Hispanic Whites were typically prescribed more opioids than minority counterparts across individual payment methods (Fig. 1). Non-Hispanic Whites consistently received more opioids in ED visits than non-Hispanic Blacks throughout all payment methods. Non-Hispanic Whites were prescribed more opioids than Hispanics when Medicaid or self-payment was used, but not when private insurance or another payment method was used.

### Clinical factors

**Fig. 2:**
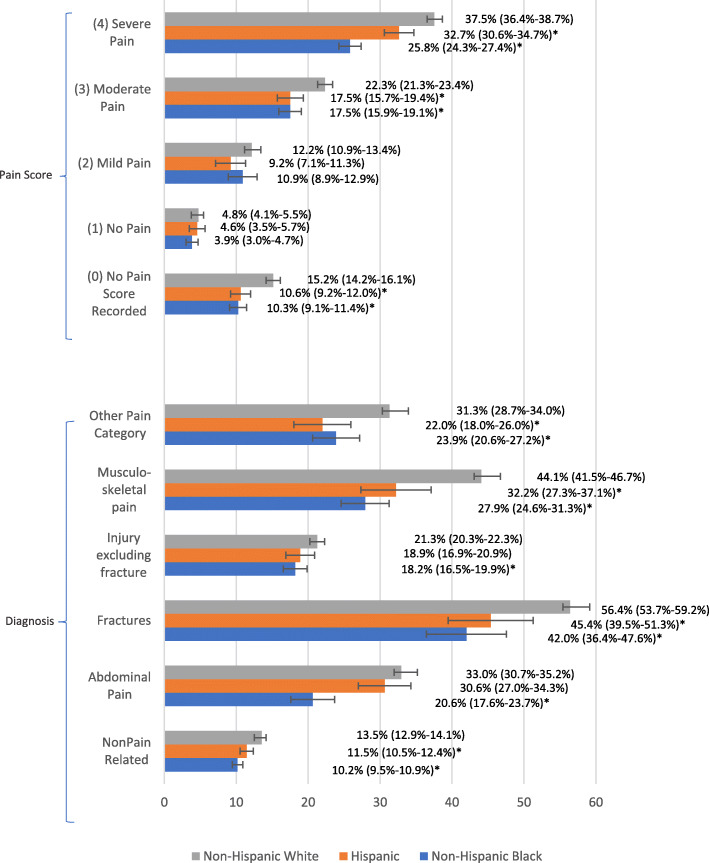
Opioid Prescription Rates by Race and Pain Diagnosis ^a^. a: All analyses accounts for the complex sampling design of NHAMCS. Tests are run at 95% Confidence intervals (*P* < 0.05). * denotes statistical significance when compared to non-Hispanic White

Assessing receipt of an opioid in the ED based on race/ethnicity within individual pain diagnoses revealed variable opioid prescribing (Fig. 2). For patients complaining of musculoskeletal pain, which included arthritis/joint pain, pelvic pain, back pain, and neck pain, receipt of an opioid was reported the most in ED visits with non-Hispanic White patients compared to both minority groups. A similar pattern is seen in visits reporting fractures, other pain, and non-pain related complaints. For ED visits associated with abdominal pain or an injury excluding fracture, non-Hispanic Whites were more likely to receive opioids than non-Hispanic Blacks, but not Hispanics.

### Multivariate analyses: logistic regression

The results of the backwards stepwise regression analysis resulted in a model of best fit that retained all loaded factors (Table 2). Race/ethnicity, age, sex, region, payment method, pain diagnosis, and pain score all showed statistically significant adjusted odds ratios (aORs). For race/ethnicity, receipt of an opioid in the ED was 32% less likely to be reported among non-Hispanic Blacks, with an aOR of 0.68 (95% CI, 0.602–0.769) compared to non-Hispanic Whites. Additionally, Hispanics were 20.2% (aOR of 0.798, 95% CI, 0.697–0.914) less likely to have received an opioid compared to non-Hispanic Whites.

**Table 2:** Odds ratio of opioid prescription by race and pain score based on logistic regression analysis^a^

	(Intercept)	aOR^c^	CI 95^b^	*P*-value
		0.017	0.014–0.021	< 0.001
Race/Ethnicity	Non-Hispanic White	–	–	–
	Hispanic	0.798	0.697–0.914	< 0.001
	Non-Hispanic Black	0.680	0.602–0.769	0.001
Pain Score	Pain Score 1 (No Pain)	–	–	–
	Pain Score 2 (Mild Pain)	2.107	1.728–2.568	< 0.001
	Pain Score 3 (Moderate Pain)	4.019	3.388–4.769	< 0.001
	Pain Score 4 (Severe Pain)	7.775	6.521–9.271	< 0.001
	Pain Score 0 (No Score Reported)	2.592	2.187–3.073	< 0.001
Pain Diagnosis	No Pain Diagnoses	–	–	–
	Abdominal Pain	2.226	1.973–2.510	< 0.001
	Fractures	7.578	6.464–8.883	< 0.001
	Injury excluding fracture	1.551	1.383–1.740	< 0.001
	Musculoskeletal Pain	3.130	2.703–3.625	< 0.001
	Other Pain Diagnoses	2.029	1.734–2.375	< 0.001
Region	Northeast	–	–	–
	Midwest	1.536	1.271–1.856	< 0.001
	South	1.904	1.612–2.250	< 0.001
	West	2.318	1.877–2.863	< 0.001
Payment Method	Private Insurance	–	–	–
	Medicaid, CHIP, State	0.808	0.740–0.883	< 0.001
	Other Payment Method	0.889	0.777–1.018	0.088
	Self-Pay	1.054	0.945–1.176	0.343
Sex	Male	–	–	–
	Female	0.860	0.783–0.944	0.001
Adolescent Age	11–14	–	–	–
	15–17	1.500	1.354–1.662	< 0.001
	18–21	2.452	2.249–2.672	< 0.001

^a^All analyses accounts for the complex sampling design of NHAMCS

^b^Tests are run at 95% Confidence intervals (*P* < 0.05)

^c^adjusted odds ratio

Logistic regression analysis comparing receiving an opioid in the ED between races/ethnicities within reported pain scores demonstrated that non-Hispanic Blacks were less likely to have received opioid reported than non-Hispanic Whites, with an aOR of 0.738 (95% CI, 0.601–0.906, *p* = 0.004) at a pain score of 3 and an aOR of 0.580 (95% CI, 0.500–0.672, *p* < 0.001) at a pain score of 4 (Table 3). Hispanics were also less likely than non-Hispanic Whites to have received an opioid reported at pain scores of 3 and 4 (aOR = 0.739 (95% CI, 0.578–0.945, *p* = 0.01) and aOR = 0.807 (95% CI, 0.685–0.951, *p* = 0.011), respectively). For visits with an unknown pain score, non-Hispanic Whites were 1.56 times (95% CI, 1.27–1.92, *p* < 0.005) more likely to receive an opioid compared to non-Hispanic Blacks. (Table 3).

**Table 3:** Odds ratio of opioid prescription by race and pain score based on logistic regression analysis^a^

Race/ethnicies	Pain Score	aOR^c^	CI 95^b^	*P*-value
Intercept		0.050	0.041–0.061	< 0.001
Non-Hispanic White	1	–	–	–
Non-Hispanic Black	1	0.803	0.550–1.171	0.245
Hispanic	1	0.963	0.620–1.496	0.866
Intercept	2	0.138	0.116–0.166	< 0.001
Non-Hispanic White	2	–		–
Non-Hispanic Black	2	0.885	0.625–1.252	0.489
Hispanic	2	0.734	0.515–1.046	0.087
Intercept	3	0.287	0.256–0.323	< 0.001
Non-Hispanic White	3	–		–
Non-Hispanic Black	3	0.738	0.601–0.906	0.004
Hispanic	3	0.739	0.578–0.945	0.016
Intercept	4	0.601	0.550–0.656	< 0.001
Non-Hispanic White	4	–		–
Non-Hispanic Black	4	0.580	0.500–0.672	< 0.001
Hispanic	4	0.807	0.685–0.951	0.011

^a^All analyses accounts for the complex sampling design of NHAMCS

^b^Tests are run at 95% Confidence intervals (*P* < 0.05)

^c^adjusted odds ratio

## Discussion

Overall, non-Hispanic Whites were more likely to receive an opioid compared to non-Hispanic Blacks and Hispanics. In this study, we found evidence of persistent racial disparities in the receipt of opioid agonists in the ED over time, with non-Hispanic Whites receiving opioids more frequently than non-Hispanic Blacks during both evaluated timeframes. Additionally, we found that racial/ethnic disparities persisted across many clinical and nonclinical factors. Evaluating the impact of race/ethnicity on the receipt of opioid agonists across different adolescent age groups, results consistently showed non-Hispanic Whites were more likely to have received an opioid agonist while in the ED. Although overall prescribing rates increased with age, non-Hispanic Whites were more likely to have received an opioid agonist across all age groups. This implies that disparities are not age-dependent; however, the trend does suggest the magnitude of disparity increases with age among adolescents when comparing non-Hispanic Blacks and non-Hispanic Whites. Further evaluation of this trend is necessary to more fully understand the impact of age on the likelihood of receiving an opioid while being treated in an ED between adolescents of these two races. The initial review of this data suggests that late adolescent Non-Hispanic Blacks likely have seen the greatest degree of bias when considering treatment for pain disorders within the ED.

When evaluating the effect of sex, the results suggest female non-Hispanic Blacks were least likely to receive an opioid, while male non-Hispanic Whites had the greatest likelihood. When comparing opioid prescriptions across males and females of the same race/ethnicity, there was no difference except for approximately a 20% reduction in receipt of an opioid between non-Hispanic Black females and males. The results suggest an amplified disparity in the treatment of pain in female non-Hispanic Blacks compared to their male counterparts not seen in non-Hispanic Whites and Hispanics. This sex-based difference deserves further research to evaluate the intersection of sex and race in this specific population. To date, no published research has evaluated within race sex/gender differences in the treatment of adolescent pain.

Within region racial/ethnic disparities consistently showed non-Hispanic Blacks being less likely to have opioid use than non-Hispanic Whites, except in the West region. The largest within region difference between races/ethnicities was seen in the Northeast, with non-Hispanic Whites being 2.2 times more likely than Hispanic partients and 1.7 times more likely than non-Hispanic Black patients to receive an opioid in the ED. These results suggest and regional aspect of racial/ethnic disparaties that likely require specific evaluation within individual regions. Additionally, the results of the study suggest racial/ethnic differences in receiving an opioid based on method of payment. Non-Hispanic White self-pay participants were 2.2 times more likely to have received an opioid compared to non-Hispanic Black Medicaid participants. This highlights the importance of ensuring consistent treatment regardless of payment method or race/ethnicity.

When examining the impact of clinical variables, disparities were observed within same pain diagnoses and within pain intensity scores of moderate (3 out of 4) and severe (4 out of 4). Among adolescents with fractures, non-Hispanic Whites were 1.34 times more likely to have received an opioid than non-Hispanic Blacks and 1.24 times more likely than Hispanics. These disparities were even more pronounced in musculoskeletal pain and abdominal pain. Although severity of fractures and pain related to individual diagnoses can vary dependent on the clinical situation, the substantial reduction in the receipt of an opioid among non-White participants raises significant concern of discrimination in the treatment of pain, regardless of individual clinical factors.

The results of this study provide a more thorough evaluation of the role of race/ethnicity on the likelihood of receiving an opioid to treat pain in adolescent patients within EDs. Previous research has been limited on finding when clinical and demographic factors are accounted for, race/ethinicity disparities persist [7–9]. This evidence further supports the presence of racial-bias of prescribers when treating pain conditions within the ED and the need to develop more robust treatment guidelines and prescribing policies related to the use of opioids in the treatment of pain in adolescents. The current lack of such guideline or universal prescribing policies may too easily allow for the introduction of bias into prescribing decisions.

To our knowledge, this is the first study to conduct an analysis of the role of race/ethnicity on the receipt of opioid agonists among adolescents while accounting for potential contributing factors. Previous studies evaluating trends in opioid use among pediatric patients have reported differences in opioid use based on race/ethnicity [3, 7, 8]; however, those studies did little to evaluate the impact of other nonclinical demographic factors and clinical factors. Additionally, previous research has often combined the receipt of opioids with non-narcotic pain medications, such as, acetaminophen and non-steroidal inflammatory drugs [9, 13]. Although the use of nonopioid pain medications can be considered as an alternative treatment in some clinical situations, the use of such agents should not differ based on patient race or ethnicity.

### Limitations

Limitations of this study include its retrospective nature and the general limitations of using a cross-sectional data set. Owing to this limitation, we can only report the findings of the analysis and are not able to draw conclusions about causality. Additionally, other factors may need to be further explored to understand their role in opioid use patterns, including the impact of prescriber specific factors, health system factors, and changes to standards of practice. For example, patient-provider concordance (i.e. patients seeing providers who are of the same race/ethnicity) has been demonstrated to have an affect on a pediatric patient’s chances of receiving an opioid and was not evaluated in the current study [32]. In addition, a number of comorbidities may affect the choice of drug for pain management and are not accounted for in this presented study. Lastly, it is important to note that race and ethnicity reporting collected was based on health record recording, which are often practitioner defined and not reported directly by patients. Although this may have resulted in incorrect race and ethnicity assignment, the focus of this paper is the impact race and ethnicity has on practitioner decisions regarding the use of opioids in the ED. This suggests practitioners’ perceptions of patient race and ethnicity is as important as a patient’s self identification of race and ethnicity in answering the objectives of the study.

## Conclusion

The finding of this study suggests racial/ethnic disparities in the receipt of opioids in EDs in the U.S. persisted from 2006 to 2016. The likelihood to recieve an opioid was greatest among non-Hispanic Whites and lowest in non-Hispanic Blacks. When accounting for various patient demographic factors (region of care, payment method, sex, and age) and clinical factors (pain diagnosis and pain score), the racial and ethnic disparities remained present. Previous research has exposed the racial and ethnic differences in opioid use, but, to date, this study is the first to evaluate the impact of multiple patient specific factors to determine if non-race/ethnicity driven factors could explain the inconsistencies in care. The findings of this study further support the existence of racial and ethnic biases in opioid prescribing trends. Of particular concern is the significant difference reported in opioid use in female non-Hispanic Blacks as compared to both their male counterparts and non-Hispanic White and Hispanic females. In addition, the increased degree of racial/ethnic inconsistencies reported by certain regions raises concern over the prescribing patterns reported in those areas. Lastly, the existence of significant variability in the use of opioids across different races/ethnicities while holding constant clinical factors suggest more efforts are needed to create evidence-based treatment recommendations for the use of opioids in adolescents to help improve the consistency in care.

## Supplementary Information

**Additional file 1.**

**Additional file 2.**

## Data Availability

This data is publicly available, which can be found at the following site: https://www.cdc.gov/nchs/ahcd/ahcd_questionnaires.htm

## Abbreviations

aOR: Adjusted odds ratio
CDC: Center for Disease Control
CI: Confidence interval
ED: Emergency department
NCHS: National Center for Health Statistics
NHAMCS: National Hospital Ambulatory Medical Care Survey
